# Peer Texting to Promote Quitline Use and Smoking Cessation Among Rural Participants in Vietnam: Randomized Clinical Trial

**DOI:** 10.3389/ijph.2024.1606941

**Published:** 2024-04-08

**Authors:** Rajani S. Sadasivam, Catherine S. Nagawa, Jessica G. Wijesundara, Julie Flahive, Hoa L. Nguyen, Celine Larkin, Jamie M. Faro, Kavitha Balakrishnan, Duc Anh Ha, Cuong Kieu Nguyen, Anh Vuong, Phuong Thu Phan, Quyen Phi Li Pham, Jeroan J. Allison, Thomas Karr Houston

**Affiliations:** ^1^ University of Massachusetts Chan Medical School, Worcester, MA, United States; ^2^ Department of Population and Quantitative Health Sciences, University of Massachusetts Chan Medical School, Worcester, MA, United States; ^3^ Department of Social and Behavioral Sciences, Harvard T.H. Chan School of Public Health, Boston, MA, United States; ^4^ Division of General Internal Medicine, Massachusetts General Hospital, Boston, MA, United States; ^5^ Ministry of Health (Vietnam), Hanoi, Vietnam; ^6^ Institute of Population, Health and Development (PHAD), Hanoi, Vietnam; ^7^ Bach Mai Hospital, Hanoi, United States; ^8^ School of Medicine, Wake Forest University, Winston-Salem, NC, United States

**Keywords:** smoking cessation, LMICs, texting, Quitlines, mhealth

## Abstract

**Objectives:** We tested an adapted version of an effective U.S.-based peer-texting intervention to promote Quitline use and smoking cessation among rural participants in Vietnam.

**Methods:** We conducted a two-arm randomized trial with participants recruited at four rural community centers. The intervention included peer messages sent for six months that promoted Quitline use and smoking cessation. Additionally, biweekly two-way text messages assessed participants’ interest in Quitline referral and current smoking status. Comparison participants received only the bi-weekly text message assessment of their current smoking status. At six months, we assessed Quitline use and smoking cessation. Smoking cessation was assessed using the 7-day point prevalence question and verified with a carbon monoxide breath monitor (<=6 ppm).

**Results:** Among 750 participants, the intervention had higher Quitline verified use (18%, 95% CI 0.14, 0.22) than comparison (1%, 95% CI .2, 2, *p* < 0.0001). Carbon-monoxide-verified smoking cessation did not differ between the two groups. However, intervention (28.3%, 95% CI) and comparison (28.1%, 95% CI) participants had substantial rates of carbon monoxide cessation at 6 months (both 28%).

**Conclusion:** Our study highlighted the promise of texting interventions to extend tobacco control efforts in Vietnam.

## Introduction

Tobacco use is associated with over 8 million yearly deaths [1, 2]. Mostly, these deaths occur in low- and middle-incomes countries (LMICs), where approximately 80% of people who smoke live [2]. Despite the World Health Organization (WHO) Framework Convention on Tobacco Control (FCTC) mandate for the treatment of tobacco use and dependence in LMICs, there is limited evidence for effective tobacco interventions, including texting interventions, in LMICs [3]. Texting interventions are valuable due to the widespread use of texting in LMICs. While systematic reviews in high-income countries have noted texting intervention effectiveness compared with no intervention or attention control [4, 5], the evidence for LIMCs is of low quality [3].

Vietnam is an LMIC with high smoking rates in men (44%) and lower in women (1%) [6]. It is estimated that over 85% of these men smoke daily [7]. Smoking-related diseases place a considerable economic burden on Vietnam (an estimated 0.97 of the total GDP in 2011) [8, 9]. Smoking cessation is emphasized in the Joint Annual Health Review (2014) published by the Vietnam Ministry of Health [10]. The country is a party to the World Health Organization (WHO) Framework Convention for Tobacco Control. Tobacco taxes were used to create a Tobacco Control fund and with these funds the Vietnam Ministry of Health has established two Quitlines (modeled after U.S. Quitlines) to support tobacco control. These Quitlines are staffed by certified tobacco treatment specialists (e.g., registered nurses and public health professionals) who have been trained in principles of motivational interviewing, tobacco risks, and evidence-based tobacco cessation strategies summarized in the 2008 U.S. Treating Tobacco Use and Dependence guidelines including the 5As (Ask, Advise, Assess, Assist, and Arrange) and the 5Rs (Relevance, Risks, Rewards, Roadblocks, and Repetition) [11]. Unfortunately, these Quitlines are underused, with one estimate suggesting they conduct as few as 25 daily calls. The Vietnam Ministry of Health is interested in methods to promote the use of these Quitlines in Vietnam. As texting is almost ubiquitous in Vietnam [12], it may be a viable option to promote Quitline use and smoking cessation. However, texting has not been tested in Vietnam for promoting Quitline use and smoking cessation.

Thus, we tested the M2Q2 texting intervention, an adaptation of an effective U.S.-based texting intervention for people in Vietnam who smoke [13–17]. The intervention used peer text messages that are pre-written advice messages written by other people in Vietnam who smoke [11]. Messages “in a smoker’s own words” enhance homophily, a feeling of similarity between the message writer and the message reader [15]. Peer messages, a form of vicarious learning, can influence self-efficacy [18]. The messages were motivational and promoted the use of the Bach Mai Quitline (one of the two Quitlines in Vietnam) and nicotine replacement therapy (NRT), which could be requested through the Quitline [11].

This paper presents the results of our randomized controlled trial that tested the M2Q2 intervention among rural Vietnamese people who smoke. We hypothesized that intervention would increase i) Quitline and NRT use, ii) smoking cessation self-efficacy, and iii) smoking cessation rates.

## Methods

### Study Design

**FIGURE 1:**
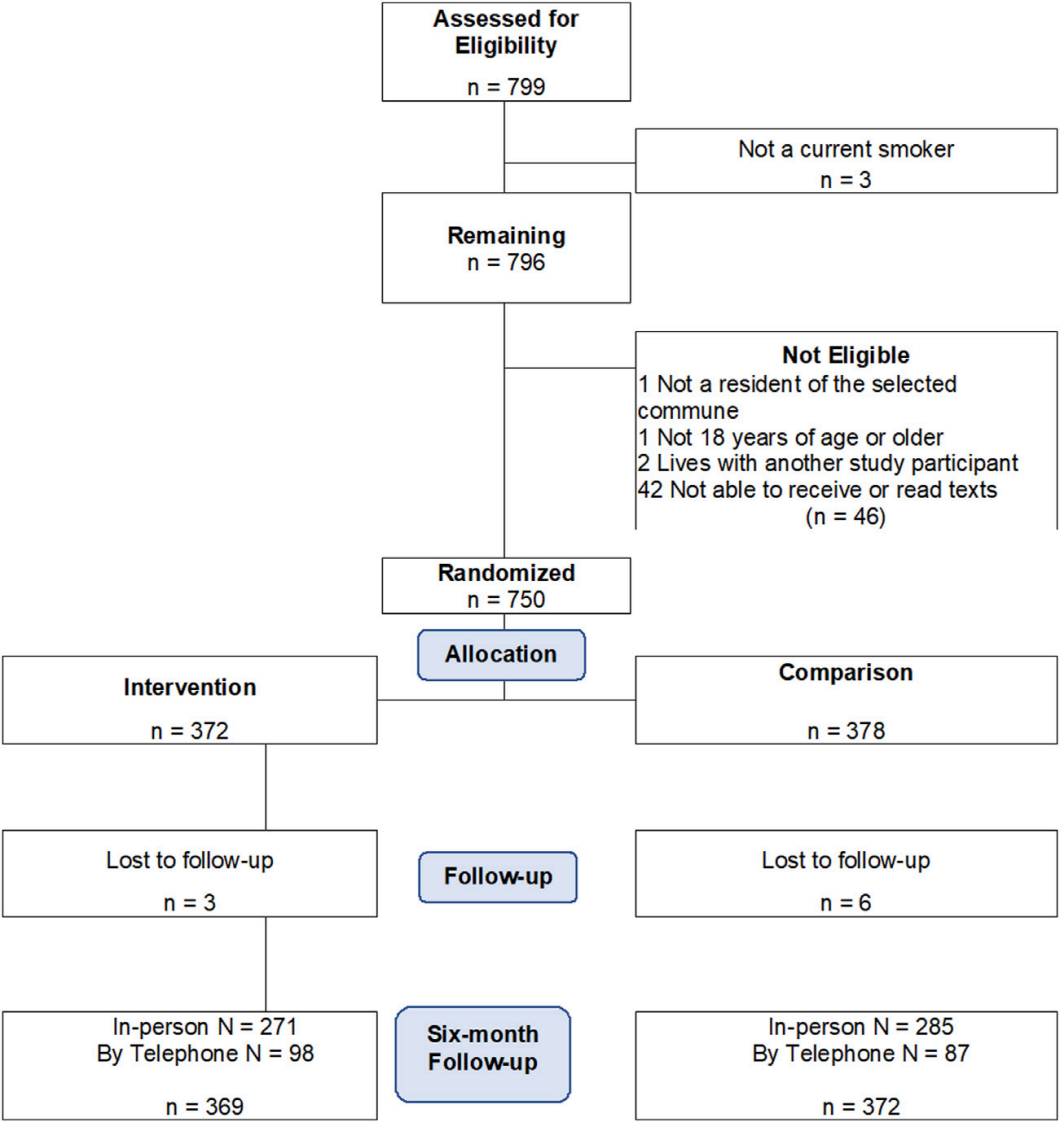
Flow diagram showing allocation of participants into study arms of a text based smoking cessation randomized intervention conducted among rural Vietnamese people who smoke (mHealth Messaging to Motivate Quitline Use and Quitting (M2Q2): RCT in rural Vietnam, Vietnam, 2017-2022).

We conducted a two-arm randomized controlled trial among participants in Hung Yen, a rural province northern Vietnam, between November 2018 and April 2021 (Figure 1). Participants were randomized and followed for 6 months [11].

### Participants, Inclusion and Exclusion Criteria

In Vietnam, the healthcare system is organized into four levels. The lowest level (the communes) contains community health centers, responsible for primary healthcare, outpatient services, and routine health delivery services. The four communes selected for this study satisfied the following criteria: (1) had a community health center with a medical doctor; (2) were not currently participating in other smoking cessation studies; and (3) had a minimum distance of 12 km from the other study communes to minimize contamination. Participant inclusion criteria included: (1) a resident of the selected commune; (2) current smoker; (3) able to receive texts and read text (literate); (4) not cognitively impaired; (5) not a participant who helped develop the motivational texts used in the intervention; and (6) not a family member of another participant in the study.

Community Health Workers (CHWs) advertised the study at community health centers. During monthly recruitment events, study staff screened interested individuals for eligibility, obtained informed consent, and completed a baseline survey before randomization. Participants received a mobile telephone credit for participating or a basic phone that could be used for texting if they did not own a mobile phone.

### Intervention and Comparison Arms

#### Intervention Condition

The intervention included text messaging and NRT provision. Participants received three types of text messages. The first type was one-way peer text messages sent daily in the first week and two messages per week for the next 25 weeks of their participation. We created new peer messages written by people who currently or recently smoked in Vietnam and had used the Bach Mai Quitline. We also adapted peer messages from those developed in U.S.-based texting studies by translating and having these same participants review and rewrite the messages to increase relevance to the target population [15]. The peer messages included motivational messages, tips, and strategies to quit smoking (e.g., tips to manage cravings) and encouraged Quitline use. The second type was a two-way message sent every 2 weeks asking participants whether they were interested in being referred to the Quitline. Quitline counselors then proactively called and enrolled interested participants in counseling sessions. We trained the Quitline counselors in best practices for counseling people who smoke (e.g., topics included motivational interviewing, pharmacotherapy, and behavioral interventions for smoking cessation). The third type was a two-way message assessing whether the participants had smoked recently, followed by tailored feedback encouraging the participants toward their smoking cessation goals, sent bi-weekly in alternate weeks to the Quitline messages. We have detailed our procedure for adapting the peer messages and the Quitline training protocol [11].

Both intervention and comparison participants could receive NRT at no cost from the communes by requesting NRT during their Quitline calls (although Quitline use was not promoted in comparison participants). However, due to high enthusiasm among the participants, many of the communes distributed the NRT among all the participants without adhering to the study protocol.

#### Comparison Condition

Participants did not receive peer messages. They received only the bi-weekly two-way assessment question assessing whether they had smoked recently. However, they did not receive tailored feedback based on their responses. We included these assessment messages to maximize blinding to the randomization group since we were promoting the study as a texting study. Quitline use was not promoted in comparison participants; however, participants were not restricted from Quitline use if they found it via other means.

### Data Collection and Main Outcomes

All survey instruments were translated into Vietnamese by a certified translator. Baseline data included demographics and smoking-related behaviors (e.g., level of addiction and readiness to quit). At 6 months, Quitline and NRT use was assessed via Quitline and commune site-collected data respectively. Self-efficacy was assessed at baseline and follow-up using the Smoking Self-Efficacy Questionnaire (SEQ-12) [19]. We assessed the 7-day point prevalence smoking cessation using the question (Do you currently smoke tobacco [smoked even 1 puff of tobacco in the last 7 days].). We also assessed other forms of tobacco use by presenting popular options of tobacco as checkboxes and an open textbox. Biochemical verification was conducted for those who self-reported as quitters, and those with greater than 6 parts per million (ppm) of carbon monoxide were classified as smokers.

### Qualitative Exit Interviews

We conducted qualitative interviews with participants (*n* = 30) who had completed the six-month follow-up and explored the use of text messages, Quitline, NRT, and the effect of COVID-19 on their tobacco use. Study staff generated a list of potential participants based on the commune of residence and smoking status and mailed them a fact sheet describing the qualitative interview. During phone calls, verbal consent was obtained, and interviews were scheduled. Participants were reimbursed for their interview participation (USD 15). Interviews were in-person, audio-recorded, transcribed, and translated into English.

### Sample Size and Power

We calculate power with the comparison cessation rate at 10%. Based on our prior trials, we have detected a 9% difference in intervention and comparison, comparing the messaging system with a robust, website-only comparison (as we have a minimal control in this study, we estimated the difference may be greater). With a sample of 600 participants, we were powered to detect a 10% difference between groups (alpha at 0.05) with 91% power. We recruited 750 participants estimating 15%–20% attrition.

### Randomization

Participant allocation to study arms was based on a permuted block scheme in which treatment assignments were made within blocks so that numbers assigned to each treatment arm are equal after filling a block. Blocks of various sizes (2, 4, and 6) were used in random order to facilitate allocation concealment. Randomization was stratified by commune site. Participants and the staff conducting the six-month outcome evaluation were also blinded to allocation condition.

### Data Analysis

The independent variable was the randomization condition for all analyses. For assessing Quitline contact and NRT use, we reported proportions and tested the differences in proportions between groups using Fisher’s exact test. The comparison of mean SEQ-12 scores between groups was conducted using a linear mixed model accounting for clustering within commune. To calculate smoking cessation, we first conducted the analysis using the 7-day point prevalence question. Logistic regression accounting for clustering within commune using a random effects model was used to report the odds of smoking cessation. We report both self-report and biochemically verified odds. Since several participants reported waterpipe use, we repeated the smoking cessation assessment by including waterpipe use in addition to the 7-day point prevalence question. An alpha level of 0.05 was used as a criterion to determine statistical significance. All analyses were conducted using SAS software (version 9.4; SAS Institute, Cary, NC).

#### Qualitative Analysis

We used Rapid Qualitative Analysis, an efficient method of qualitative analysis when pre-set questions are being explored (e.g., acceptability of the texting system), to extract information from each interview and to compile findings across interviews in a series of domains [20–22], an efficient method of qualitative analysis when pre-set questions are being explored (e.g., acceptability for the texting system).

## Results

The study was conducted among 750 current smokers recruited through four community health centers, with 372 being randomly assigned to the intervention group and 372 randomly assigned to the intervention group and 378 randomly assigned to the comparison group (Figure 1). Our overall follow-up rate was 98.8%. Carbon monoxide biochemical verification of smoking cessation had a lower follow-up rate (*n* = 178/365 of those who self-reported quitting smoking, 48.8%) due to COVID-19-related protocol changes, as it was deemed unsafe to use the carbon monoxide devices during the pandemic. In addition, a carbon monoxide device failure during one of the follow-up events affected the data collection of 19 participants.

Sex was not an eligibility criterion because rates of smoking among women are very low in Vietnam, and therefore, the sample was 100% male. There were no baseline differences in participant’s demographics (Table 1) or smoking-related behaviors (Table 2). There were no baseline differences in characteristics between those who completed the carbon monoxide verification and others.

**TABLE 1:** Demographic and socio-economic characteristics of participants randomized to a text-based smoking cessation intervention (mHealth Messaging to Motivate Quitline Use and Quitting (M2Q2): RCT in rural Vietnam, Vietnam, 2017-2022).

Participant characteristics	Intervention	Comparison	*p*-value
	*N* = 372	*N* = 377^a^	
Age, mean (SD)	42.7 (12.5)	42.7 (12.6)	0.96
Education Level			
Primary school	27 (7.3)	28 (7.4)	0.94
Completed secondary school	165 (44.4)	175 (46.4)	
Vocational college/college/university or above	65 (17.5)	61 (16.2)	
Completed high school	115 (30.9)	113 (30.0)	
Marital Status			
Divorced or widowed/Separated/Single	49 (13.2)	52 (13.8)	0.80
Married/A member of an unmarried couple	323 (86.8)	325 (86.2)	
Employment			
Self-employed	82 (22.0)	95 (25.2)	0.43
Farmer	95 (25.5)	79 (21.0)	
Paid Work	155 (41.7)	165 (43.8)	
Other	40 (10.8)	38 (10.1)	
Number of adults in household, mean (SD)	3.2 (1.2)	3.3 (1.4)	0.29
Number of children in household, mean (SD)	1.5 (1.3)	1.3 (1.2)	0.08
Past 12 months: were worried or stressed about having enough money?			
Never/Rarely	195 (52.4)	211 (56.0)	0.22
Sometimes	84 (22.6)	66 (17.5)	
Usually/Always	93 (25.0)	100 (26.5)	
Number of Comorbidities			
None	176 (47.3)	173 (45.9)	0.42
One comorbidity	104 (28.0)	121 (32.1)	
Two or more comorbidity	92 (24.7)	83 (22.0)	
How would you describe your own health?			
Excellent/Very Good	87 (23.4)	74 (19.6)	0.61
Good	266 (71.5)	281 (74.5)	
Fair	17 (4.6)	20 (5.3)	
Poor	2 (0.54)	2 (0.53)	
In the last 6 months, did you have to stay overnight in the hospital for any reason?			
Yes	20 (5.4)	19 (5.0)	0.84
No	352 (94.6)	358 (95.0)	

^a^ One participant did not complete the baseline survey and subsequently withdrew from the study.

**TABLE 2:** Smoking characteristics of participants randomized to a text-based smoking cessation intervention (mHealth Messaging to Motivate Quitline Use and Quitting (M2Q2): RCT in rural Vietnam, Vietnam, 2017-2022).

Participant characteristics	Intervention	Comparison	*p*-value
	N = 372	N = 377^a^	
Number of cigarettes consumed per day, mean (SD)	13.5 (8.9)	13.2 (11.2)	0.71
Age when first smoked tobacco; mean (SD)	18.7 (6.1)	18.6 (5.6)	0.80
Number of years: daily tobacco use: mean (SD)	23.9 (13.4)	23.7 (13.8)	0.83
How soon after you wake up do you first smoke tobacco?			
Within 5 min	153 (41.1)	135 (35.8)	0.30
6–30 min	117 (31.5)	116 (30.8)	
31–60 min	21 (5.6)	25 (6.6)	
After 60 min	81 (21.8)	101 (26.8)	
Do you smoke other tobacco products?			
Yes	198 (53.2)	202 (53.6)	0.92
No	174 (46.8)	175 (46.4)	
Other tobacco products smoke			
Water pipes	196 (52.7)	202 (53.6)	0.81
Have you ever tried an “e-cigarette,” even just one time?			0.16
Yes	77 (20.7)	63 (16.7)	
No	295 (79.3)	314 (83.3)	
Does your workplace have any rules about smoking tobacco?			0.62
Yes	88 (23.7)	95 (25.2)	
No	284 (76.3)	282 (74.8)	
How much money per week do you currently spend on tobacco products?			
0–10,000 VND	47 (12.6)	66 (17.5)	0.10
10,000–20,000 VND	18 (4.8)	30 (8.0)	
20,000–30,000 VND	46 (12.4)	40 (10.6)	
30,000–40,000 VND	50 (13.4)	41 (10.9)	
>40,000 VND	211 (56.7)	200 (53.1)	
Quit attempt in past 12 months			0.57
Yes	210 (56.5)	205 (54.4)	
No	162 (43.6)	172 (45.6)	
Before being contacted for this survey, had you ever heard of the Bach Mai Quitline?			0.32
Yes	58 (15.6)	69 (18.3)	
No	314 (84.4)	308 (81.7)	
Besides yourself, does anyone who lives in your home currently smoke tobacco?			0.78
Yes	111 (29.8)	109 (28.9)	
No	261 (70.2)	268 (71.1)	
Baseline smoking self-efficacy SEQ-12, mean (SD)	32 (9.9)	33 (9.9)	0.10

^a^ One participant did not complete the baseline survey and subsequently withdrew from the study.

### Quitline Use

The intervention group had a significantly higher proportion of Quitline-verified use than the comparison group (proportion: 0.18, 95% CI 0.14, 0.22 vs. proportion: 0.01, 95% C 0.002, 0.042, *p* < 0.0001; Table 3). Quitline users had a mean age of 43 (Standard Deviation SD = 12) years, most had completed secondary school (39%) or high school (33%), and the majority were married or in a relationship (92%). About 41% smoked their first cigarette within 5 min of waking up, and 35% smoked within 6–30 min of waking. About 27% of them lived with someone who also smoked tobacco. The mean number of cigarettes smoked per day was 14 (SD = 8.9). It took a mean of 9.4 (SD = 13.2) days from when participants indicated they were interested in talking with the Quitline to their first call. Quitline users completed a mean of 5.3 (SD = 3.4) calls.

**TABLE 3:** Quitline use and self-efficacy at six-month follow-up for participants randomized to a text-based smoking cessation intervention (mHealth Messaging to Motivate Quitline Use and Quitting (M2Q2): RCT in rural Vietnam, Vietnam, 2017-2022).

Quitline use	Intervention (*n* = 369)	95% CI of proportion	Comparison (*n* = 372)	95% CI of proportion	*p*-Value
	Proportion		Proportion		
Quitline verified use	0.18	(0.14, 0.22)	0.01	(0.002, 0.02)	<.0001^a^
Self-reported any Quitline use	0.25	(0.21, 0.3)	0.02	(0.009, 0.04)	<.0001^a^
**Nicotine Replacement Therapy (NRT) use**	**proportion**	**95% CI of proportion**	**proportion**	**95% CI of proportion**	***p*-value**
Commune verified NRT use	0.19	(0.15, 0.23)	0.18	(0.14, 0.22)	0.78
Self-reported NRT use	0.25	(0.21, 0.3)	0.24	(0.20, 0.29)	0.82
**Smoking Self-Efficacy SEQ-12**	**mean (SD)**	**95% CI of mean**	**mean (SD)**	**95% CI of mean**	***p*-value**
	43 (10.6)	(42, 44)	43 (10.2)	(42, 44)	0.88^b^

^a^ Fisher’s exact test for the *p*-value.

^b^ F-test *p*-value from linear regression model accounting for clustering within commune.

### Nicotine Replacement Therapy (NRT) Use

There were no differences in NRT use between the two groups (Table 3).

### Self-Efficacy

Smoking self-efficacy increased from baseline in both groups (32–43 in the intervention group, and 33 to 43 in the comparison group) with no significant difference between groups at 6 months (Table 3).

### Smoking Cessation

Overall, 28% of participants had quit at 6 months. There were no differences in the odds of carbon monoxide-verified smoking cessation (Odds Ratio: 0.99, 95% CI 0.57, 1.7; Table 4). Waterpipe use was reported by 105 (28%) of intervention and 103 (28%) of comparison participants at follow-up. After including waterpipe use, we estimated that 23% of participants had quit at 6 months and the odds of carbon monoxide-verified smoking cessation was 0.92 (95% CI 0.51, 1.7).

**TABLE 4:** Smoking cessation at six-month follow-up for participants randomized to a text-based smoking cessation intervention (mHealth Messaging to Motivate Quitline Use and Quitting (M2Q2): RCT in rural Vietnam, Vietnam, 2017-2022).

	Intervention	Comparison	OR (95% CI of proportion) for intervention vs. Comparison^a^	*p*-Value
	Proportion	Proportion		
Carbon monoxide verified smoking cessation	(*n* = 326)	(*n* = 339)		
7-day point prevalence biochemically verified	0.28	0.28	0.99 (0.57, 1.7)	0.97
7-day point prevalence and waterpipe use biochemically verified	0.22	0.24	0.92 (0.51, 1.7)	0.68
Self-report smoking cessation	(*n* = 369)	(*n* = 372)		
7-day point prevalence only	0.52	0.50	1.1 (0.82, 1.5)	0.58
7-day point prevalence and waterpipe use	0.37	0.36	1.1 (0.65, 1.7)	0.74

^a^ Accounting for clustering within commune.

### Qualitative Findings

Most participants endorsed that the text messages were understandable and acceptable in tone. While participants liked the frequency of the text messages, some reported that receiving them during work hours when they did not have their phone was inconvenient. Some participants reported ignoring or deleting texts because they thought they were spam.

Participants were positive about their Quitline counselors and found them caring and “friendly like a family member.” Other participants felt that they were able to quit on their own and did not need the Quitline or NRT. A community physician usually prescribed NRT. While most found the NRT helpful, some participants reported experiencing side effects from NRT (e.g., itchy throat, nausea, and gastrointestinal problems) but did not report stopping NRT for these reasons.

The COVID-19 pandemic occurred during the trial, and participants reported differing effects on their smoking habits. Some participants reported smoking less because they were at home more, had less money to spend on cigarettes, were wearing masks, or were more aware of the health effects of COVID-19 on people who smoke. Conversely, some participants reported smoking more during the pandemic, also because they were at home more, were bored, or because they believed that smoking would protect them against COVID-19. For example, one participant stated: “If I smoke, I think that COVID will be afraid of me.”

## Discussion

The M2Q2 texting intervention significantly increased Quitline use among participants from a rural province in northern of Vietnam. While the intervention did not increase smoking cessation compared with the comparison group, the quit rates in both groups (28%) were remarkable. For comparison, the “Treating Tobacco Use and Dependence: 2008 Update” reported an estimated quit rate of 24.7% (21.0–28.4) for intensive in-person interventions greater than eight sessions [23]. A recent clinical trial that tested the efficacy of varenicline reported a 7-day point prevalence of 29.0% in the varenicline arm, compared to 6% in the placebo arm [24]. Our findings also revealed self-efficacy increased by greater than one standard deviation from baseline to follow-up for both groups, another remarkable temporal trend. Further, there were notable differences in cessation rates between the self-report and carbon-monoxide-verified cessation, which suggests that future trials in low- and middle-income countries like Vietnam should carefully consider how to measure cessation.

Our study findings pose important questions for Vietnam, a country that has only recently begun addressing tobacco use. The texting intervention increased Quitline use, showing it may be a viable option in Vietnam. People in rural parts of Vietnam may not have been exposed to messages promoting Quitline use and thus may have been more willing to accept the referral to the Quitline in our study. The two prior studies that tested the use of texting for increasing Quitline use were both conducted in the United States and reported varied results, possibly due to differences in the frequency of the messages promoting Quitline use [25, 26]. Future studies could experiment with the number and framing of messages to determine the optimal approach to motivate Quitline use.

It was remarkable that both groups achieved high quit rates and increased self-efficacy, suggesting that Vietnam may be at a pivotal transitional time in their fight against tobacco use. A high proportion of people in Vietnam who smoke have reported making recent quit attempts (37.5%) compared to other countries [27], but may simply not have had access to tobacco cessation programs given the low reach of current tobacco efforts. Our study suggests that even minimal exposure to a tobacco cessation program, such as our comparison (i.e., participating in a tobacco cessation study and text assessment of current smoking), could have a high impact on reducing the tobacco use rate in Vietnam. Investing in such tobacco programs that deliver minimal exposure to tobacco cessation and could be implemented to have a broad reach may considerably benefit Vietnam. These could include training providers or CHWs to deliver brief interventions (such as the 5As) or to continue to improve the texting program as discussed below.

In addition, despite its lack of success in increasing tobacco cessation rates in our study, we posit that Vietnam must continue to invest in more intensive forms of tobacco control, like Quitlines, to ensure that tobacco control services are distributed fairly and equitably. This is critical to avoid the challenges faced by the U.S. tobacco control efforts, which, although effective in reducing overall tobacco use, have led to segments of the population being left behind. Highlighted below are potential areas of improvement in the intervention components based on our results.

The Vietnam Quitline modeled on the U.S. (e.g., frequency and number of calls) may not be optimized for people in Vietnam. Another study that evaluated the Quitline services in Vietnam also reported high relapse rates despite high satisfaction among participants [28]. Additional research on the specific challenges of the people in Vietnam who smoke may be needed. We found the intervention did not increase self-efficacy compared to the comparison, and self-efficacy has been shown as a mediator of tobacco cessation [29]. Future studies could investigate additional approaches to increasing self-efficacy, such as supporting the practice of short-term quit attempts designed to help the participant succeed, which can lead to increased knowledge, self-efficacy, and potentially cessation [30].

Similar to another study, there were no additive effects of the combination of text messaging and Quitline use [31]. Our text messages were designed to be independent of Quitline use as we anticipated only a subset would use the Quitline. Text messages that reinforce the messages of the counselors may more effectively promote synergism between the Quitline and the text messages, and future trials should test this integrated approach. Participants’ feedback on our text messaging intervention was varied and suggested that further refinement of the messages, including providing choices for the timing of the messages, may increase the intervention’s effectiveness.

There is debate on whether light-touch interventions (such as M2Q2) with minimal direct contact with participants, as opposed to interventions such as individual counseling, needs biochemical verification of smoking cessation. Some argue that the social desirability in light-touch interventions is low and that needing biochemical verification may reduce the generalizability of the sample (as only the more motivated subset might participate) and the study’s feasibility [32, 33]. The high disagreement in our results between the self-report and the carbon-monoxide verified results may indicate that the social desirability in countries like Vietnam is higher and that even light touch interventions in these countries would benefit from biochemical verification.

Our study is the first trial to test a texting intervention among rural participants of a LMIC, as other such studies have been conducted in urban populations [3]. Our follow-up rate of 98% was also high. We recruited rural participants in northern Vietnam, who seldom have the chance to join research studies, through community health workers. This recruitment approach may have increased their appreciation and motivation for engaging with our study. However, we had limitations in our study. Although we did not exclude participants based on their motivation to quit smoking, we acknowledge that those who participated in the study may have had higher motivation levels than the general population. Despite informing the participants during the baseline session about the specific cell phone number that would send them text messages, we could not estimate how many ignored the messages as spam. As noted, a protocol deviation reduced our ability to detect differences in NRT use. We also did not collect additional details on NRT use, including dosage and duration. The COVID-19 pandemic reduced our ability to conduct biochemical verification with all participants. We also collected only minimal data about how the COVID-19 pandemic may have impacted our participants’ smoking behaviors.

### Conclusion

Our trial adds to the growing literature on mobile interventions for tobacco cessation in LMICs. Our participants were from rural areas who were underrepresented in prior trials in LMICs. Our results showed that texting is a viable strategy to increase the use of Quitline services in Vietnam, while also raising important implications for future tobacco policy in Vietnam, such as whether an intervention that provides minimal exposure to tobacco cessation, like our comparison, could be used for increasing reach and impact of tobacco interventions. Our next steps are to explore additional ways to increase the effectiveness of the Quitline and the texting system, such as incorporating strategies to promote self-efficacy, closer integration of the text messages with the Quitline messages and providing additional flexibility in the timing of the messages.
